# Omega-3 polyunsaturated fatty acids selectively inhibit growth in neoplastic oral keratinocytes by differentially activating ERK1/2

**DOI:** 10.1093/carcin/bgt257

**Published:** 2013-07-26

**Authors:** Zacharoula Nikolakopoulou, Georgios Nteliopoulos, Adina T. Michael-Titus, Eric Kenneth Parkinson

**Affiliations:** Centre for Clinical and Diagnostic Oral Sciences, Institute of Dentistry, Barts and the London School of Medicine and Dentistry, Queen Mary University of London, Turner Street, London E1 2AD, UK,; ^1^Department of Haematology, Imperial College, Commonwealth Building, Du Cane Road, London W12 0NN, UK and; ^2^Centre for Neuroscience and Trauma, Blizard Institute for Cell and Molecular Science, Barts and the London School of Medicine and Dentistry, Queen Mary University of London, 4 Newark Street, London E1 2AT, UK

## Abstract

The long-chain omega-3 polyunsaturated fatty acids (n-3 PUFAs)—eicosapentaenoic acid (EPA) and its metabolite docosahexaenoic acid (DHA)—inhibit cancer formation *in vivo*, but their mechanism of action is unclear. Extracellular signal-regulated kinase 1/2 (ERK1/2) activation and inhibition have both been associated with the induction of tumour cell apoptosis by n-3 PUFAs. We show here that low doses of EPA, in particular, inhibited the growth of premalignant and malignant keratinocytes more than the growth of normal counterparts by a combination of cell cycle arrest and apoptosis. The growth inhibition of the oral squamous cell carcinoma (SCC) lines, but not normal keratinocytes, by both n-3 PUFAs was associated with epidermal growth factor receptor (EGFR) autophosphorylation, a sustained phosphorylation of ERK1/2 and its downstream target p90RSK but not with phosphorylation of the PI3 kinase target Akt. Inhibition of EGFR with either the EGFR kinase inhibitor AG1478 or an EGFR-blocking antibody inhibited ERK1/2 phosphorylation, and the blocking antibody partially antagonized growth inhibition by EPA but not by DHA. DHA generated more reactive oxygen species and activated more c-jun N-terminal kinase than EPA, potentially explaining its increased toxicity to normal keratinocytes. Our results show that, in part, EPA specifically inhibits SCC growth and development by creating a sustained signalling imbalance to amplify the EGFR/ERK/p90RSK pathway in neoplastic keratinocytes to a supraoptimal level, supporting the chemopreventive potential of EPA, whose toxicity to normal cells might be reduced further by blocking its metabolism to DHA. Furthermore, ERK1/2 phosphorylation may have potential as a biomarker of n-3 PUFA function *in vivo*.

## Introduction

Oral squamous cell carcinomas (SCCs) are the sixth most common cancers worldwide (1). SCCs of the aerodigestive tract account for ~10% of the malignancies in the developed world and are very expensive to treat (2), so there is a strong health economic argument for reducing the number of advanced cases, particularly in risk groups, such as former smokers. A major problem is that these SCCs arise from a generalized field of abnormal mucosa that can evolve in widely separated areas of the aerodigestive tract. Second field or second primary cancers are a common cause of relapse (3,4), suggesting that cost-effective chemopreventive strategies could be a novel adjunct to conventional therapies. Several studies suggest that the long-chain omega-3 polyunsaturated fatty acids (n-3 PUFAs)—eicosapentaenoic acid (EPA) and docosahexaenoic acid (DHA)—have significant chemopreventive and therapeutic potential against cancer (reviewed in ref. 5). Dietary intake of n-3 PUFAs is associated with a lower risk of several cancers, including SCC (6), and inhibits both the growth (7) and metastasis (8) of animal tumours and xenografts (8). Topically applied EPA also inhibits both the initiation and promotion phases of mouse epidermal tumorigenesis without affecting cell proliferation (9). The fat-1 transgenic mouse, which produces a higher level of n-3 fatty acids endogenously, is resistant to several types of cancer (10–12). The beneficial effects of n-3 PUFAs in the treatment and prevention of cancer may involve a multitude of mechanisms, but the accessibility of aerodigestive tract and epidermal SCCs to therapeutic aerosols or gels makes it an attractive model to test the therapeutic and prophylactic potential of n-3 PUFAs. The safety and tolerability of these compounds have already been documented in other clinical indications (13). However, the effects of n-3 PUFAs on malignant cells and their premalignant or normal counterparts have not been compared previously.

There is considerable evidence that both DHA and EPA can induce apoptosis in several cancer cell lines (14,15) but whether this is the full explanation for their anticancer activity is not clear, and there is little consensus on their molecular mechanism of action, which appears to be pleiotropic. Some studies link the tumour-suppressive action of n-3 PUFAs with the mitogen-activated protein kinase pathway and either the activation or inactivation of its components with the induction of apoptosis (16,17) and growth arrest (18,19), suggesting that there may be tissue- or cancer-specific mechanisms of n-3 PUFAs action. In particular, as regards extracellular signal-regulated kinase 1/2 (ERK1/2), some studies showed that n-3 PUFAs promote apoptosis in cancer cells by reducing the levels of p-ERK1/2 (20–23). Traditionally, the activation of ERK1/2 is linked to cell survival and proliferation (24). However, recent studies demonstrated that this is not always the case, and activation of ERK could actually cause apoptosis or growth arrest (25–28). At higher doses, n-3 PUFAs have been reported to induce the production of reactive oxygen species (ROS) (29) and c-jun N-terminal kinase (JNK) phosphorylation and induce apoptosis (30) or growth arrest (19).

We report here that both DHA and EPA induce apoptosis and growth arrest in human normal and neoplastic keratinocytes from the epidermis and oral cavity. However, in particular, EPA, at a lower dose, inhibited the growth of premalignant and malignant keratinocytes more than the growth of normal keratinocytes, whereas DHA was less selective. The growth inhibition at the selective low doses of EPA required occupancy of the epidermal growth factor receptor (EGFR) and was associated with a sustained activation of ERK1/2, which did not occur in non-neoplastic keratinocytes at the same dose. PI3 kinase was not activated in parallel. Therefore, our results suggest that n-3 PUFAs may exert some of their anticancer effects by inducing over-stimulation of ERK1/2 or a signalling imbalance downstream of the EGFR pathway. Our results also suggest that n-3 PUFAs or their derivatives may have potential in the prevention or reduction of aerodigestive tract and epidermal SCC.

## Materials and methods

### Cell lines

SCC-13 and SCC-25 are tumorigenic keratinocyte lines—epidermal (facial epidermis) and oral (tongue), respectively (31)—and SVHFK is an origin of replication-defective SV40 mutant virus-transformed epidermal keratinocyte line that is not tumorigenic at early passage but becomes so after extensive passaging (32); it can, thus, be considered premalignant. NHEK-131 (Invitrogen, Paisley, UK) and HEK-127 are normal foreskin epidermal keratinocyte lines. The five cell lines were maintained in keratinocyte basic medium (Cambrex-Lonza, Walkersville, MD) supplemented with bovine pituitary extract (0.03mg/ml), human EGF (0.1ng/ml), insulin (5.0g/ml), hydrocortisone (0.5g/ml) and antibiotics/antimycotics GA-1000 (gentamicin at 50g/ml and amphotericin B at 50ng/ml) to make keratinocyte growth medium. Two oral normal NHOK-810 and NHOK-881, two non-neoplastic immortal OKF6/TERT-1 (33) and OKF4/cdk4R/p53DD/TERT (34) and three oral dysplasia cell lines D17, D19 and D20 (35) were maintained in keratinocyte-SFM (1×) medium supplemented with human recombinant 0.2ng/ml EGF 1–53 and 25 µg/ml bovine pituitary extract (Invitrogen), 10 µg/ml penicillin and streptomycin (Cambrex-Lonza) and 0.4mM calcium chloride (33). Cells were maintained in a humidified atmosphere of 5% CO_2_/95% air (epidermal) and 10% CO_2_/90% air (oral) at 37°C.

The cells were disaggregated with 0.1% trypsin (Worthington, Lakewood, NJ)/0.01% ethylenediaminetetraacetic acid (Sigma–Aldrich, Gillingham, UK) in phosphate-buffered saline when they reached ~50% confluence.

### Chemicals

5-8-11-14-17 EPA and 4-7-10-13-16-19 DHA (NU-CHEK, Elysian, MN) were purchased as free fatty acids in the form of pure oil and were diluted in ethanol under nitrogen at a stock concentration of 0.5M. The stock was aliquoted in dark-coloured glass vials with screw tops (Agilent Technologies, Wokingham, UK) to protect from light and oxidation and stored at −20°C for up to 6 months. The antioxidant n-*tert*-butyl-α-phenylnitrone (PBN) was obtained from Sigma (Poole Dorset, UK) and added to the growth medium to give final concentrations between 50 and 1mM. The caspase inhibitor Q-VD-OPh was from Calbiochem (La Jolla, CA). The MEK inhibitor U0126 was from Cell Signaling Technology (Danvers, MA) (data not shown) and AZD6244 was from Selech Chemicals (Houston, TX). The EGF receptor inhibitor AG1478 was from Invitrogen. The EGFR-blocking antibody [EGFR Mouse anti-Human Monoclonal (Azide-free) (225) Ab] was from LifeSpan Biosciences (Seattle, WA). The inhibitors were added in the culture for 1.5 h and the blocking antibody for 4 h before the addition of EPA and DHA. After 2 h since the n-3 PUFAs were added, the lysates were obtained.

### MTT assay

The cell viability was determined using a colorimetric 3-(4,5-dimethylthiazol-2-yl)-2,5-diphenyltetrazolium bromide (MTT) assay (Sigma). The cells were seeded at 24-well plates and treated. Medium was replaced with 1 ml serum-free medium containing 0.5mg/ml MTT solution and incubated for 1 h, then the MTT solution was removed, and 0.5 or 1ml of dimethyl sulphoxide was added to dissolve the formazan dye. Triplicates of 200 µl aliquots from each well were transferred to a 96-well plate, and absorbance was read at 540 nm using a plate reader.

### Annexin-V apoptosis assay

Cells were trypsinized combined with any cells in suspension and pelleted. The resuspended cells were then incubated at room temperature for 15min in 500 µl of Annexin-V Binding Buffer (Becton Dickinson, Oxford, UK) containing 4 µl/ml of Annexin-V-FLUOS (Roche Diagnostics Ltd, Burgess Hill, UK) and 200ng/ml of 4′,6-diamidino-2-phenylindole (DAPI) viability dye (Sigma). The samples were analysed on a flow cytometer (LSR II BD Biosciences) collecting 10–20 000 events.

### ^3^H-Thymidine incorporation assay

Cell proliferation was measured by incorporation of tritiated thymidine (^3^H-TdR). Cells were seeded at 4 × 10^2^ cells/ml/well into sterile 96-well flat-bottom tissue culture plates (Fisher Scientific, Edmonton, Alberta, Canada) and treated with 3 µM of EPA and DHA, as described for the MTT assay. Cell cultures were pulsed with 0.5 µCi/well of ^3^H-TdR (Amersham, UK) and incubated for 18 h. Cells were then harvested onto filters (Wallac Perkin Elmer) using a 96-well cell harvester (TOMTEC Harvester 96/Mach 3M). Scintillation fluid (Wallac) was added to the filter before detection of radioactive energy on a beta counter (Wallac). The results of the incorporated thymidine, in triplicate, were expressed in counts per minute. Counts per minute were then expressed as a percentage of the PUFA-untreated control.

### Preparation of lysates and western blotting

Cells were washed twice in phosphate-buffered saline at 4°C and lysed in radioimmunoprecipitation assay lysis buffer (1% NP-40, 0.1% sodium dodecyl sulphate, 50mM Tris, pH 7.3 and 150mM NaCl) (all from Sigma) and protease (cOmplete cocktail tablets) and phosphatase (PhosSTOP cocktail tablets) inhibitors (both from Roche Diagnostics) for 20 min on ice and centrifuged at 15 000 r.p.m. for 20 min at 4°C. Supernatant protein concentrations were determined by the DC protein assay (Bio-Rad, Hemel Hempstead, UK), and 10–35 µg of protein was boiled for 5min at 100°C in sodium dodecyl sulphate sample buffer (5×, 0.3M Tris–HCl, pH 6.8, 10% sodium dodecyl sulphate, 50% glycerol, 20% 2-mercaptoethanol, 0.25% bromophenol blue; all from Sigma) before storage at −80°C.

Lysates were run on 4–12% NuPAGE Novex Bis-Tris gels under denaturing and reducing conditions (Invitrogen) at 130V and transferred to 0.45 µm Immobilon PVDF membranes (Millipore, Watford, UK) by a wet transfer system (Invitrogen). Membranes were blocked in 5% low fat milk (Marvel, Bristol, UK) in Tris-Buffered Saline and Tween 20 (TBS-T) (1M Tris, pH 8.0, 5M NaCl and 0.05% Tween 20) (all from Sigma) for 1 h. Primary antibody was added in 5% milk in TBS-T or 5% bovine serum albumin (PAA, Pasching, Austria) in TBS-T, according to the antibodies manufacturer’s instructions, incubated overnight at 4°C and then washed with TBS-T. The membrane was incubated in appropriate horseradish peroxidase-conjugated secondary antibody in 5% milk in TBS-T at room temperature for 1h and then washed again with TBS-T and then visualized by chemiluminescence (Amersham ECL Plus) on Amersham Hyperfilm ECL (both from GE Healthcare Life Sciences).

### Antibodies

Cleaved caspase 3, cleaved caspase 8, cleaved caspase 9 (Asp330), caspase 8, caspase 9, phospho-p44/42 MEK (Thr202/Tyr204) (phospho-ERK1/2), total AKT, phospho-p90RSK (rabbit polyclonals; Cell Signaling), anti-Akt/PKB[pS^473^] (rabbit polyclonal; Biosource-Invitrogen) and caspase 3 (goat polyclonal from R&D systems) were all used at 1:1000 dilution. P44/42 MEK (ERK1/2) (mouse monoclonal; Cell Signaling) was used at 1:2000. RSK1/2/3 and EGFR (both from Cell Signaling) and phospho-EGFR (BD Transduction Laboratories) antibodies were used at 1:1000 and 1:500, respectively. Glyceraldehyde 3-phosphate dehydrogenase (GAPDH) mouse monoclonal and α-tubulin rabbit polyclonal antibodies (both from Abcam) were used at 1:5000 and 1:2000, respectively. Secondary antibodies were horseradish peroxidase-conjugated anti-rabbit and anti-mouse IgG goat polyclonal antibodies (Pierce, Thermo-Scientific) and an anti-goat IgG mouse monoclonal antibody (Sigma) used at 1:2500, 1:3000 and 1:10 000, respectively.

### Detection of ROS and oxidative damage and antioxidant treatment

The 2′,7′-dichlorodihydrofluorescein diacetate (Calbiochem-Merck, Nottin gham, UK) cell-permeable fluorogenic probe, that detects the ROS and NO, was used to determine the overall oxidative stress in the cells. Moreover, dihydroethidium (Invitrogen), which is a superoxide indicator, was used to confirm oxidation (data not shown). After the cells were treated with DHA and EPA for 16 h, they were incubated with 1 µM of 2′,7′-dichlorodihydrofluorescein diacetate (DCF) or 5 µM of dihydroethidium and 250ng/ml of DAPI for 30min in 37°C. The fluorescent intensity of the dye uptake was measured by a flow cytometer (LSR II BD Biosciences), collecting 10–20 000 events. The HFF cell line, which is a human fetal skin fibroblast cell line with low ROS levels, was used as negative control, whereas cells treated with 100 µM of *tert-*butyl hydroperoxide (which causes oxidative damage), for just 2 h at 37°C, were used as positive controls.

The 8-hydroxy-2′-deoxyguanosine antibody (anti-8-oxo-dG, Clone 2E2) from Trevigen (Gaithersburg, MD) was used to detect oxidative damage by immunocytochemistry according to the supplier’s protocol. The cells were treated with DHA and EPA for 16 h. Cells treated with 100 µM *tert-*butyl hydroperoxide at 37°C for 2 h were used as positive controls. The cells were visualized with a Leica DM5000 epifluorescence microscope under the ×40 objective lens and ×100 oil emersion lens and analysed with the Metamorph Imaging Software (Sunnyvale, CA).

SCC-25 cells were treated with the antioxidants PBN 600 and 800 µM or α-tocopherol (40 µM) (both from Sigma) before the addition of the n-3 PUFAs and the MTT assay. PBN was shown to reduce oxidation at the concentrations used (36).

### Statistical analysis

The non-parametric Wilcoxon Mann–Whitney Rank test and the one-way analysis of variance (ANOVA) followed by *post* *hoc* Bonferroni test were used and performed via the SPSS statistical software (version 17; Chicago, IL). A *P* value <0.05 was considered significant.

## Results

### The n-3 PUFAs, EPA and DHA selectively inhibit the growth of premalignant and malignant keratinocytes compared with their normal or immortalized counterparts

**Fig. 1.**
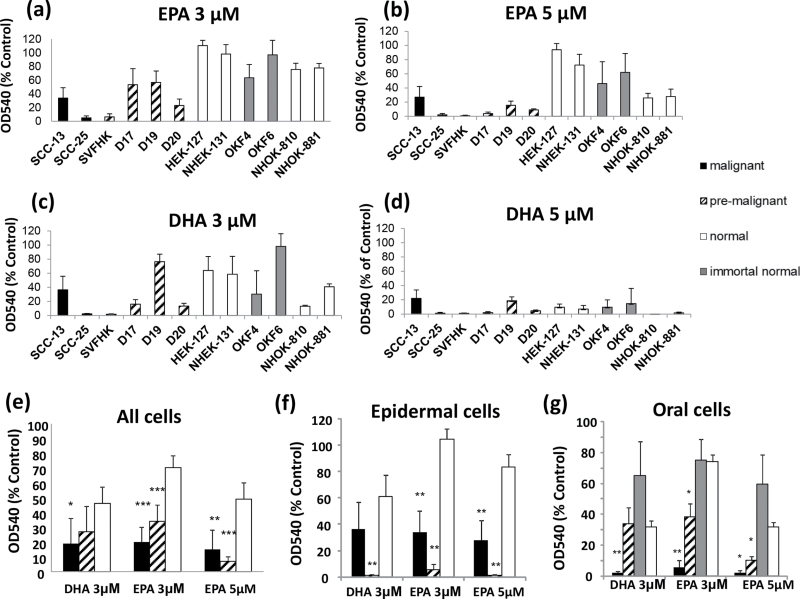
The effect of n-3 PUFAs on the growth of normal, premalignant and malignant keratinocytes. The growth of the keratinocytes was measured by the MTT viability assay 4 days after the addition of the n-3 PUFAs at the indicated doses. The effect of 3 µM EPA (**a**), 5 µM EPA (**b**), 3 µM DHA (**c**) and 5 µM DHA (**d**) on the growth of the malignant (black), premalignant (hatched), normal (white) and immortal normal (grey bars) keratinocytes is shown, as determined by the MTT assay. The results are the means of three or more experiments ± standard error of the mean (SEM). (**e**) An overview of the means ± SEM of the MTT assay for the malignant (black), premalignant (hatched) and normal keratinocytes (white) averages. The overview for the epidermal keratinocytes (**f**) and the oral keratinocytes (**g**) is shown, where the immortal normal oral lines are included (grey bars). Significantly different from the mean value of normal keratinocytes (*P < 0.05; **P < 0.01; ***P < 0.001 as measured by the Mann–Whitney *U*-rank test). The malignant cells include SCC-25 (oral) and SCC-13 (epidermal). The premalignant cells include the three oral dysplasias (D17, D19 and D20) and the epidermal SVHFK. The normal cells include the normal oral lines (NHOK-810 and NHOK-881), the epidermal lines (HEK-127 and NHEK-131) and the immortal normal oral lines OKF6/TERT-1 and OKF4/cdk4R/p53DD/TERT.

Figure 1 shows that both DHA and EPA inhibit the growth of premalignant and malignant keratinocytes more than their normal or immortalized counterparts, as assessed by the MTT assay. EPA was far more selective than DHA causing 2.5-fold (premalignant) to 3.5-fold (malignant) more growth inhibition than in their normal counterparts at a dose of 3 µM and 4-fold (malignant) to 6-fold (premalignant) at 5 µM (a highly significant difference), as compared with DHA, which caused only 1.5-fold (premalignant: not significant) to 2-fold (malignant) more growth inhibition at a dose of 3 µM. DHA was not selective at 5 µM (Figure 1d). In the epidermal lines studied, the SV40-transformed line SVHFK, which has premalignant properties (32), was more sensitive than the SCC line, SCC-13 and both were more sensitive than the two normal epidermal lines NHEK-131 and HEK-127 (Figure 1a–c and f). All three oral dysplastic lines (35) were less sensitive than their malignant counterpart SCC-25 at 3 µM but more sensitive than the two normal oral keratinocyte lines tested and also more sensitive than two normal oral keratinocyte lines immortalized by telomerase (Figure 1a and b).

### The increased sensitivity of the neoplastic keratinocytes lines is independent of immortalization, telomerase activation and p53 and/or p16^INK4A^ inactivation

Normal oral keratinocytes lines immortalized by ectopic telomerase expression (OKF6/TERT-1 and OKF4/cdk4R/p53DD/TERT) were more resistant to n-3 PUFAs growth inhibition than normal keratinocytes (Figure 1a–c). Previous studies have shown that OKF6/TERT-1 line also has reduced p16^INK4A^ (33), and OKF4/cdk4R/p53DD/TERT additionally has inactivated p53 and p16^INK4A^ (34). In contrast, although the premalignant SVHFK line would also have inactive p53 and pRb/p16^INK4A^ pathways owing to the presence of SV40 large T antigen and expresses telomerase, it would additionally harbour the oncogenic effects of the small T antigen (32) and was hypersensitive to both n-3 PUFAs, as was the mortal dysplastic D17 line. These observations suggest that as yet uncharacterized oncogenic mutations in the dysplastic and SCC lines, and not the events leading to immortalization, such as p16^INK4A^/p53/telomerase dysfunction, are connected with the increased sensitivity of these neoplastic keratinocytes to the n-3 PUFAs.

### Growth inhibition by n-3 PUFAs in keratinocytes is mediated by both apoptosis and cell cycle arrest

**Fig. 2.**
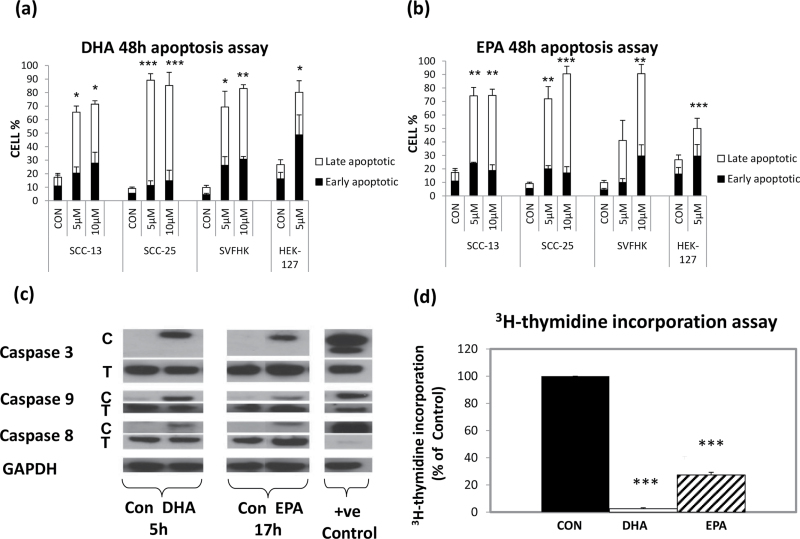
n-3 PUFAs inhibit growth by inducing apoptosis and inhibiting cell proliferation. Annexin-V assays showing the effect of (**a**) DHA and (**b**) EPA on apoptosis 48 h after adding doses of 5 and 10 µM to normal (HEK27), premalignant (SVHFK) and malignant (SCC-13 and SCC-25) keratinocytes. Early apoptotic cells are indicated by the black part of the bar, and the late apoptotic and necrotic cells (Annexin-V +ve/DAPI +ve) by the white part of the bar. The results in (**a**) and (**b**) are the means of three experiments ± SEM. Significantly different from the mean value of normal keratinocytes [*P < 0.05; **P < 0.01; ***P < 0.001 as measured by one-way ANOVA followed by *post* *hoc* Bonferroni test]. (**c**) Western blot analysis of caspases 3, 8 and 9 in SCC-25 cells treated for 5 h with DHA and 17 h with EPA 10 µM compared with the ethanol vehicle control. C = cleaved caspase; T = total caspase; GAPDH is the loading control. The positive control was an extract from normal keratinocytes NHEK treated with cisplatin for 24 h. The blots are representative of three independent experiments. (**d**) ^3^H-thymidine incorporation assay performed on SCC-25 cells treated with a dose of 3 µM DHA (white bars) and EPA (hatched bars) for 48 h compared with the vehicle control (black bars). The results are the means of six independent experiments ± SEM. SCC-25 cells were incubated in 3 µM DHA and EPA for 48 h in keratinocyte growth medium serum-free medium and ^3^H-thymidine was added 18h prior to the incorporated thymidine measurement. The values were normalized to a percentage of the untreated control, which was taken as 100%. Significantly different from the mean value of the untreated control (***P < 0.001 as measured by one-way ANOVA followed by *post* *hoc* Bonferroni test).

n-3 PUFAs have been reported to inhibit both cell proliferation (19) and induce apoptosis (17) to reduce viable cell number. Therefore, we investigated the basis for the inhibition of SCC-25 growth in more detail as it was the cell line, which showed the greatest response to n-3 PUFA-induced growth inhibition. The detection of Annexin-V, which recognizes translocated phosphatidylserine (37), was used to detect early apoptotic cells, and the simultaneous application of the DNA stain DAPI allowed the discrimination of the necrotic cells from the Annexin-V-positive cells. Supplementary Figure 1, available at *Carcinogenesis* Online, shows representative fluorescence-activated cell sorting data showing the reduced viability from 93% in the control to 14% after 5 µM DHA and 41% after 5 µM EPA. In general, DHA resulted in a higher percentage of late apoptotic cells (Figure 2a) than EPA (Figure 2b) after 48 h of treatment and caused a greater induction of total apoptosis in normal keratinocytes (3-fold versus 2-fold) at a dose of 5 µM. DHA induced apoptosis in the neoplastic cells by 4- to 9-fold and EPA by 4- to 7-fold at the same dose.

To confirm that the dead cells were indeed apoptotic, we subjected the n-3 PUFA-treated SCC-25 cells to western blot analysis and tested for the cleavage of three caspases known to mediate apoptosis. Lysates were obtained for different time points of incubation with DHA and EPA between 30min and 24h. Figure 2c shows that both caspase 8 and caspase 9, in addition to the executioner caspase 3, were cleaved within 5 h of DHA treatment and within 17 h of EPA treatment. These data support the hypothesis that n-3 PUFAs were causing growth inhibition in part by inducing apoptosis, and the activation of caspase 8, in addition to caspase 9, suggests that both the extrinsic and the mitochondrial apoptotic pathways were involved (38).

In addition, we used a ^3^H-thymidine incorporation assay to test whether n-3 PUFAs were also capable of inhibiting SCC-25 proliferation, and Figure 2d shows that this was indeed the case with DHA-treated cells showing virtually no incorporation and EPA-treated cells showing >75% reduction.

### n-3 PUFAs activate the EGFR in SCC but not in non-neoplastic keratinocytes

**Fig. 3.**
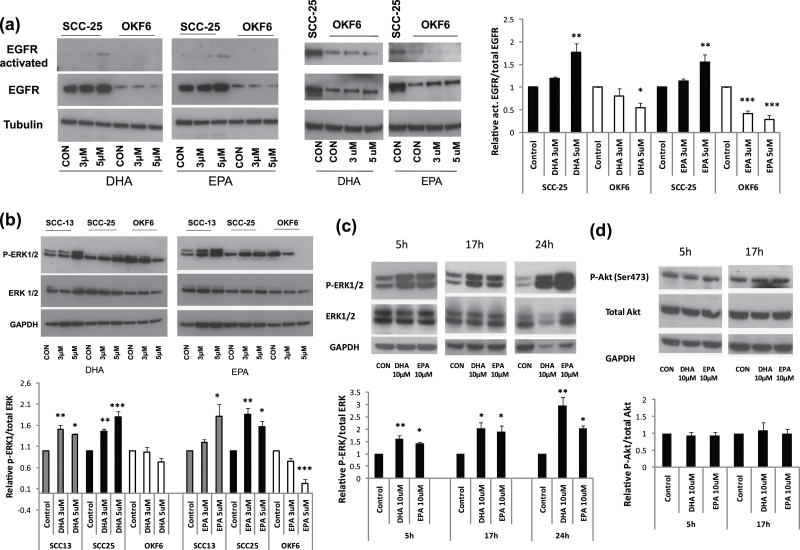
Effect of n-3 PUFAs on EGFR, ERK1/2 and Akt phosphorylation. (**a**) Western blots showing the activation (phosphorylation) of EGFR in SCC-25, but not in OKF6/TERT1 (OKF6), by DHA and EPA and also the higher constitutive levels of EGFR in SCC-25. The loading control (tubulin) is shown in the lower panel. Additionally, the overexposed blot shows the big difference between the activated EGFR in SCC-25 and OKF6 and also the decrease of EGFR activation after treatment with DHA and EPA. (**b**) Western blots showing increased phosphorylation of ERK1/2 (top panel) relative to total ERK1/2 (middle panel) in both SCC-13 and SCC-25, but not in OKF6, after the addition of 3 and 5 µM of both n-3 PUFAs for 2 h. In fact, ERK1/2 phosphorylation was reduced in OKF6 at 5 µM. The loading control (GAPDH) is shown in the bottom panel. (**c**) Western blots showing the sustained induction of ERK1/2 phosphorylation (top panel) by 10 µM DHA and EPA after 5, 17 and 24 h compared with total ERK1/2 (middle panel) and GAPDH (bottom panel). (**d**) Western blot showing an absence of any notable change in Akt phosphorylation (top panel) by the same doses of DHA and EPA after 5 and 17 h compared with total Akt (middle panel) and GAPDH (bottom panel). Representative blots and densitometry of at least three or more independent experiments (for the quantification of the results) are shown. The levels of the phosphorylated proteins after treatment with DHA and EPA are normalized with the total protein and expressed as relative values compared with the protein levels of the vehicle control. Significantly different from the mean value of the untreated vehicle controls (*P < 0.05; **P < 0.01; ***P < 0.001 as measured by one-way ANOVA followed by *post* *hoc* Bonferroni test).

Figure 3a shows that both DHA and EPA induce autophosphorylation and activation of the EGFR in SCC-25 after 2 h. However, EGFR phosphorylated levels were considerably lower in the immortal non-neoplastic OKF6/TERT-1 keratinocytes, which has much lower endogenous levels of EGFR, and in fact EGFR activation is decreased by EPA and to a lesser extent, by DHA. The increased activation of EGFR in SCC-25 relative to the non-neoplastic OKF6/TERT-1 keratinocytes suggested that activation of EGFR could be linked to the selective growth inhibition of the former, as high doses of EGF that are beneficial to normal keratinocytes inhibit SCC keratinocytes (39), and sustained ERK1/2 phosphorylation downstream is associated with growth inhibition (40). Therefore, we investigated whether ERK1/2 was differentially phosphorylated in SCC cells.

### n-3 PUFAs increase ERK1/2 phosphorylation and activation in SCC but do not activate Akt

ERK1/2 inhibition has been previously linked to n-3 PUFAs anticancer action (17,19,20). We investigated the ERK1/2 phosphorylation in the non-neoplastic immortal oral keratinocyte line OKF6/TERT-1 and two SCC lines, SCC-13 and SCC-25, from the epidermis and oral cavity, respectively. Figure 3b shows that interestingly within 2 h of the addition of 3 and 5 µM EPA and DHA, increased ERK1/2 phosphorylation is detectable in SCC-13 and SCC-25 but not in OKF6/TERT-1. In fact, ERK1/2 phosphorylation declined in n-3 PUFA-treated OKF6/TERT-1 cells, especially at the 5 µM dose in parallel with the effect of the lipids on growth (Figure 1a–d) and with the decrease of activated EGFR (Figure 3a). We did not detect increased endogenous ERK1/2 phosphorylation in SCCs, relative to their immortal but non-neoplastic counterpart. These results show that n-3 PUFAs affect ERK1/2 signalling differently in normal and malignant keratinocytes, suggesting that increased and sustained ERK1/2 phosphorylation may have a role in the selective inhibition of SCC growth by these lipids.

Figure 3c shows that within 5 h, 10 µM of both n-3 PUFAs leads to a significantly high activation of ERK1/2 in SCC-25 cells that was sustained for at least 24 h, and in other experiments, this increase in phosphorylation was detectable within 30 min (data not shown). SCC-25 cells overexpress EGFR by 10-fold and sustained activation of the MEK pathway has been implicated in the growth arrest and differentiation in neuronal cells overexpressing EGFR (40), whereas transient activation is usually associated with proliferation. As PI3 kinase has been implicated in both protecting cells against apoptosis (41,42) and senescence (a form of permanent cell cycle arrest) (43), we tested for any alterations of the levels of phosphorylation of the downstream target of PI3 kinase, Akt, following n-3 PUFA treatment. Figure 3d shows that the levels of p-AKT do not change significantly, which is consistent with a sustained ERK1/2 activation inducing apoptosis or growth arrest rather than proliferation.

### ERK1/2 phosphorylation by n-3 PUFAs requires activation of EGFR but not the caspases

**Fig. 4.**
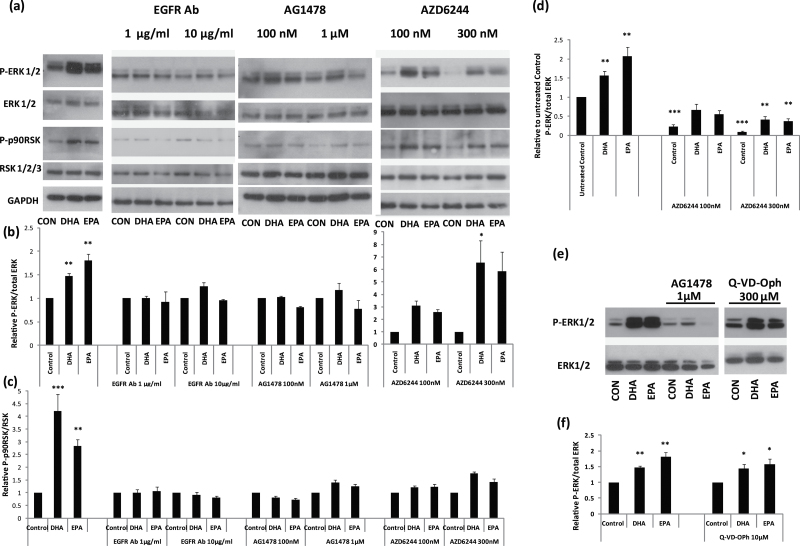
The inhibition of ERK1/2 phosphorylation and function by EGFR and MEK antagonists. (**a**) Western blots showing the effect of the indicated antagonists on ERK1/2 phosphorylation and its downstream target P-p90RSK as induced by 3 µM DHA and EPA in addition to the ethanol control (CON) 2 h after treatment. Total ERK1/2 and RSK1/2/3 as well as GAPDH served as loading controls. The EGFR neutralizing antibody (EGFR-Ab) was used at 1 and 10 µg/ml; the EGFR kinase inhibitor AG1478 was used at 100 nM and 1 µM; and the MEK inhibitor AZD6244 was used at 100 and 300 nM. (**e**) Western blot comparing the effect of AG1478 with the pan-caspase inhibitor Q-VD-Oph on ERK1/2 as induced by 5 µM DHA and EPA in addition to the ethanol vehicle control 2 h after treatment. The blots are typical of three independent experiments. The graphs show the relative to the control mean value of P-ERK/total ERK (**b** and **f**) and P-p90RSK/total RSK (**c**), and they represent the mean values of three or more western blots measured by densitometry. (**d**) The graph shows the relative mean values of the n-3 PUFA- and AZD6244-treated cells compared with the untreated vehicle control. Significantly different from the mean value of the untreated vehicle control (*P < 0.05; **P < 0.01; ***P < 0.001 as measured by one-way ANOVA followed by *post* *hoc* Bonferroni test).

As SCC-25 cells are known to possess a high density of EGFR on their cell surface, we used this line to investigate the mechanisms of n-3 PUFAs action further. Figure 4 shows that the EGFR kinase inhibitor, AG1478, and the MEK inhibitor, AZD6244 (Figure 4a and b), both inhibited the phosphorylation of ERK1/2 induced by both DHA and EPA in SCC-25 cells. To test whether occupancy of the EGFR was required for activation of ERK1/2, we treated SCC-25 cells with an EGFR-blocking antibody and showed that this was indeed the case at both doses 1 and 10 µg/ml, which also inhibited the phosphorylation of the ERK1/2 target p90 ribosomal S6 kinases (p90RSK) (Figure 4a and c). The effect of AZD6244 on n-3 PUFA-induced ERK1/2 phosphorylation seems much less than that of the EGFR inhibitors (Figure 4a and b). However, this is probably because (at least partly) it had a clear effect on the endogenous levels of ERK1/2 phosphorylation in the controls (Figure 4d). However, there is still the possibility that pathways other than MEK may mediate the activation of ERK1/2 by n-3 PUFAs. Taken together, our results suggest that the n-3 PUFAs amplify EGFR signalling through a sustained activation of the ERK/RSK pathway to cause apoptosis and growth inhibition in SCC-25 cells. As many SCCs have 5–10 times more EGFR than their normal counterparts (44), this could explain the selective effect of n-3 PUFAs on neoplastic keratinocytes.

It has been reported that ERK1/2 phosphorylation can be a survival mechanism so the consequence (rather than the cause) of apoptosis (45,46). Although the cleavage of caspases 3, 8 and 9 was much slower than the rapid phosphorylation of ERK1/2, we tested the hypothesis further by treating SCC-25 cells with both n-3 PUFAs and the pan-caspase inhibitor Q-VD-Oph (Figure 4e and f) and found no evidence for a reduction in n-3 PUFA-induced ERK1/2 phosphorylation, contrary to the results with AG1478. Therefore, we conclude that ERK1/2 phosphorylation induced by n-3 PUFAs is not a consequence of apoptosis.

### EGFR occupancy is required for growth inhibition by EPA

**Fig. 5.**
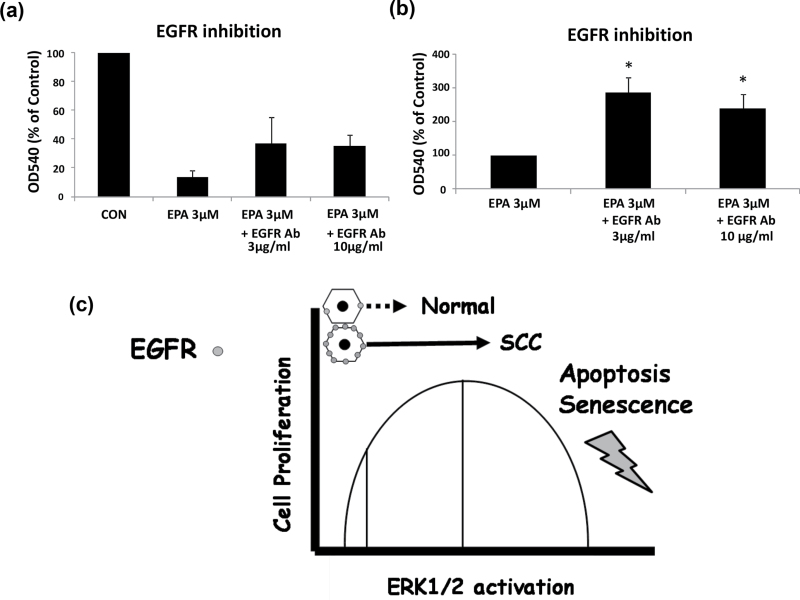
The EGFR neutralizing antibody partially rescues EPA growth inhibitory effect. (**a**) MTT viability assay showing the extent of the growth inhibition by EPA with and without two doses of the EGFR neutralizing antibody that had previously been shown to block ERK1/2 phosphorylation by EPA. The control was an isotype-matched antibody. The results are the means of four independent experiments ± SEM. (**b**) Graph showing the same data expressed relative to the effect of EPA on growth in the presence of the isotype control antibody. *P < 0.05. (**c**) Schematic representation of our model is shown. The occupancy of EGFR by ligands such as transforming growth factor-α is hypothesized to be important to the inhibition of proliferation and the induction of apoptosis by EPA, based on the effects of the EGFR-blocking antibody (a)and (b). The diagram summarizes the hypothesis that EPA selectively inhibits the growth of human SCC cells by inducing a sustained activation of ERK1/2 (solid arrows) to inhibit proliferation and induce apoptosis, whilst having the opposite (or no) effect on normal cells, which normally respond to EGFR occupancy with transient and reduced ERK activation (hatched arrow).

To test whether the increased ERK1/2 phosphorylation was essential for the growth inhibitory effect of n-3 PUFAs we examined whether the EGFR neutralizing antibody could antagonize the growth inhibitory effect of EPA and DHA in SCC-25 cells using the MTT assay. The results showed that the growth inhibitory effect of EPA (Figure 5a and b), but not DHA (data not shown), was partially reversed by the inclusion of the EGFR-blocking antibody, which inhibits n-3 PUFA-induced ERK1/2 phosphorylation (Figure 4a), from 12% of the ethanol control levels in the isotype control to around 40%. The results support the hypothesis the increased ERK1/2 activation by EPA is partially responsible for the growth inhibition of SCC-25 and that occupancy of EGFR is required for this effect (Figure 5c). The lack of an effect of the EGFR antibody on DHA-treated cells suggests that DHA activates growth inhibitory pathways in addition to those activated by EPA (see below).

### ROS generation, oxidative damage and JNK phosphorylation by n-3 PUFAs at high doses may explain the weaker selective effect of DHA compared with EPA

**Fig. 6.**
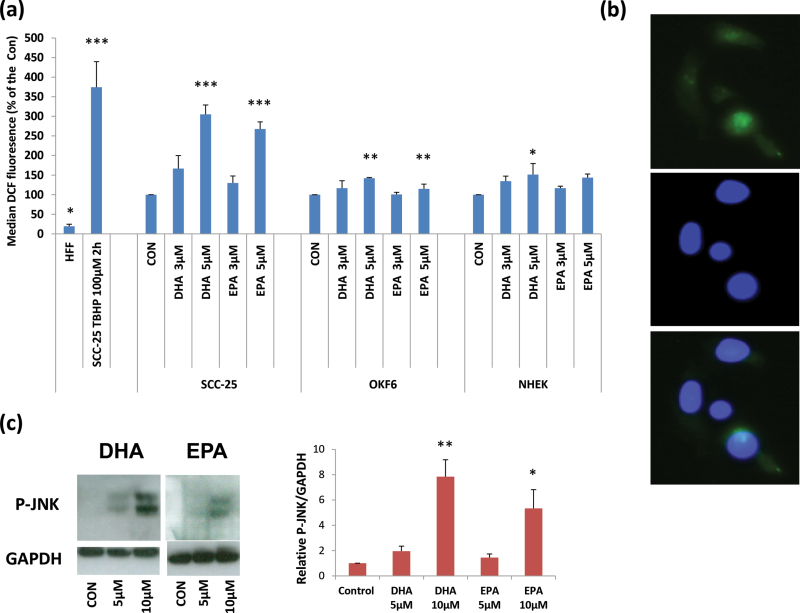
Effect of n-3 PUFAs on ROS levels and JNK phosphorylation in SCC-25 cells. (**a**) A DCF fluorescence-activated cell sorting assay showing the effect of 3 and 5 µM EPA and DHA on the ROS levels in SCC-25 cancer cells together with OKF6/TERT-1 (OKF6) and NHEK-131 (NHEK) normal keratinocytes. The results are expressed as percentages of each vehicle control. Human fetal skin fibroblasts with low ROS production served as a negative control and *tert-*butyl hydroperoxide-treated SCC-25 (TBHP) cells as a positive control and were expressed as a percentage of the SCC-25 untreated control. (**b**) Representative picture of DCF positively stained cells showing DCF staining (upper panel), nuclear staining with Hoechst 33258 (central panel) and an overlay of the two (bottom panel). (**c**) Representative western blot showing the effect of DHA and EPA at 5 and 10 µM after 2 h on JNK phosphorylation, showing an increase at both 5 and 10 µM with DHA appearing more potent than EPA. The graph summarizes the results of three western blots measured by densitometry. The results are the means of three experiments ± SEM. Significantly different from the mean value of the untreated vehicle controls (*P < 0.05; **P < 0.01; ***P < 0.001 as measured by one-way ANOVA followed by *post* *hoc* Bonferroni test).

DHA is just as specific as EPA in inducing ERK1/2 phosphorylation in SCC keratinocytes relative to their normal counterparts, yet is much less specific at inhibiting their growth, especially at 5 µM, where it has a considerable effect on normal keratinocytes (Figure 1). As n-3 PUFAs have been reported to induce the production of ROS and phosphorylate JNK at higher doses (19,30), we hypothesized that DHA might generate more ROS than EPA and, as a result, have a greater effect on normal or non-neoplastic-immortal keratinocytes. Figure 6 shows that both DHA and EPA cause oxidative stress in SCC-25 cells at doses of 5 µM and above as assessed by the fluorescence of DCF followed by fluorescence-activated cell sorting analysis (Figure 6a), dihydroethidium staining (data not shown) and the presence of 8-oxo-dG staining of the cell nuclei (Supplementary Figure 2, available at *Carcinogenesis* Online). However, EPA was less effective than DHA at inducing ROS at both 3 and 5 µM (Figure 6a), and DHA showed much more 8-oxo-dG staining at 3 µM than EPA (Supplementary Figure 2, available at *Carcinogenesis* Online) and more phosphorylation of JNK at 5–10 µM in SCC-25 cells (Figure 6c). There was lower ROS produced at the more selective dose of 3 µM that had a minimal growth inhibitory effect on normal keratinocytes (Figure 1). When ROS was measured in the normal keratinocytes NHEK-131 and OKF6/TERT-1, both n-3 PUFAs still produced ROS but less compared with SCC-25. However, the increase in ROS was statistically significant only at the dose of 5 µM.

As both EPA and DHA were shown to elicit oxidative damage and activate JNK in SCC-25 cells at doses of 5–10 µM (Figure 6; Supplementary Figure 2, available at *Carcinogenesis* Online), we tested whether the antioxidants PBN and α-tocopherol (data not shown) could reverse the growth inhibitory effects of DHA and EPA at doses of 3–10 µM, but this was not the case (Supplementary Figure 3, available at *Carcinogenesis* Online), suggesting that the induction of ROS is not the only cause of the n-3 PUFA-induced growth inhibition but may compromise its specificity.

## Discussion

Many previous studies support the hypothesis that n-3 PUFAs can inhibit the formation and progression of cancers (5,9,11,12,14), and other studies have highlighted their role in augmenting therapeutic strategies (14). However, although it is acknowledged that n-3 PUFAs may act on the cancer microenvironment for example, by inhibiting inflammation (47), the mechanism of action of this important class of lipids in tumour suppression is incompletely understood. In particular, EPA, but not its metabolite DHA, has been reported to inhibit the promotion phase of epidermal SCC development (9), rendering it a strong candidate for a chemopreventive agent of epidermal and aerodigestive tract SCC, where field cancerization is a major clinical problem (3,4). Furthermore, the accessibility of much of the aerodigestive tract and the epidermis to aerosols or gels would help deliver higher local doses.

We show here that EPA is much more selective in the growth inhibition of premalignant human oral keratinocytes than DHA, although both showed some selectivity against SCC cells. The growth inhibitory effect was a combination of inhibited proliferation and the induction of apoptosis, in agreement with Schley *et al*. (18), via both the intrinsic (caspase 9) and extrinsic pathways (caspase 8).

Interestingly, in our study, the inhibition of keratinocyte growth was associated with a rapid and sustained phosphorylation of ERK1/2 in neoplastic cell lines. The phosphorylation of ERK1/2 and partially, the growth inhibitory effect of EPA, were dependent on the occupancy and activation of EGFR. ERK1/2 phosphorylation was not accompanied by a concomitant phosphorylation of AKT indicative of increased PI3 kinase activity. This is not surprising as EGFR has been reported to enhance ERK phosphorylation downstream of MEK through dual-specificity phosphatases (48) but could result in a signalling imbalance that has previously been shown to trigger apoptosis (41,42) or senescence (43). The phosphorylation of ERK1/2 and its downstream target p90RSK, by both EPA and DHA, was antagonized by the EGFR-blocking antibody (mAb 225), the EGFR kinase inhibitor AG1478 but to a lesser extent by the MEK inhibitors, U0126 (data not shown) and AZD6244, which supports the hypothesis that ERK1/2 phosphorylation might also be increased by pathways other than MEK (48).

Activation of the ERK1/2 pathway was not inhibited by a pan-caspase inhibitor, we were able to partially rescue the growth inhibitory effects of EPA with an EGFR-blocking antibody, and the phosphorylation of ERK1/2 following EPA treatment occurred within 30 min, whereas caspase activation, as determined by the cleavage of caspase 3, took over 5 h. Therefore, the phosphorylation of ERK1/2 was not just a survival mechanism and a consequence of apoptosis as reported in certain settings (45,46).

Even though the activation of ERK is traditionally linked to cell survival and proliferation (24), several recent studies show that activation of ERK could actually cause apoptosis or cycle arrest (25–28). Wang *et al.* (49) showed that cisplatin-induced apoptosis was dependent on MEK/ERK signalling and Elder *et al.* (25) demonstrated that sustained ERK1/2 activation mediates apoptosis induction by the nonsteroidal anti-inflammatory drug NS-398 in colon cancer cells. So ERK activation effect is not as straightforward as previously thought, and it depends on the type, strength and duration of the stimulus and on the cell type (25). Therefore, an increase in ERK1/2 phosphorylation upstream of apoptosis is not without precedent.

Our data are consistent with the inhibition of cell growth by a sustained activation of ERK1/2 (40), whereas the inhibition of SCC growth by inducing a sustained activation of EGFR has been also previously reported (50). The selective inhibition of growth seen in neoplastic keratinocytes could be explained by the higher density of EGFR in these cells (44) (Figure 5c).

Some previous studies have linked the n-3 PUFA tumour-suppressive action with the mitogen-activated protein kinase pathway, although showed that n-3 PUFAs, especially DHA, can promote apoptosis by inhibiting ERK1/2 phosphorylation (17,19,20). The *fat-1* mouse shows slightly lower levels of MEK and ERK1/2 phosphorylation in normal breast tissue compared with the wild-type (17), similar to our observations on a non-neoplastic keratinocyte line. However, the status of ERK1/2 phosphorylation was not examined in *fat-1* mouse breast tumours and so this report (17) does not conflict with our new observations. Notably, and consistent with our study, another group has reported that both DHA and EPA can cause a decrease in EGFR levels in lipid rafts isolated from a human breast cancer line and that this was associated with an increase in EGFR (16) and also with apoptosis (18). However, in these studies, no link between increased EGFR and ERK1/2 phosphorylation, which leads to growth inhibition and apoptosis, was established, and no comparison with normal cells was made.

Although both n-3 PUFAs induced a rapid phosphorylation of ERK1/2 and its downstream target p90RSK, DHA produced rather more ROS and phosphorylated JNK than EPA in SCC-25, and this might explain why the EGFR-blocking antibody failed to rescue its growth inhibitory effects. Both n-3 PUFAs produced ROS in normal cells, which is statistically significant at the higher doses but generally less compared with SCC-25. A possible explanation is that they bear protective mechanisms against ROS, which might be lost in cancer cells and p53, dysfunctional in most of the neoplastic cells in this study, is known to orchestrate an antioxidant response (51). Taken together, these support the hypothesis that the generation of ROS might be one reason why some of the normal cells are dying, especially at high doses of PUFAs, and this might compromise their selective growth inhibition towards neoplastic keratinocytes. As DHA is synthesized from EPA, albeit inefficiently in humans (52), our data suggest that modifying EPA, so that it produces less DHA might reduce the formers toxicity to normal cells; this could also be achieved by inhibiting an essential enzyme, such as Δ6-desaturase (52). Even though both n-3 PUFAs generated ROS at the higher doses, caused oxidative damage and activated JNK, ROS generation was not the only mechanism of growth inhibition at these doses because the cells could not be rescued by antioxidants. However, the effects of EPA could be rescued by blocking the EGFR, albeit not completely, suggesting that several mechanisms including ROS induction may contribute to n-3 PUFAs growth inhibition of SCC-25 cells.

In conclusion, we have shown that n-3 PUFAs, and especially EPA, have a selective growth inhibitory effect on neoplastic human keratinocytes, causing both reduced proliferation and apoptosis (intrinsic and extrinsic pathway), and that this effect is partially mediated by activation of the ERK1/2 pathway through EGFR, demonstrated here for the first time. The results suggest that EPA should be considered as a chemopreventive agent for SCC, both in the context of preventing tumour recurrence from a cancerous field or its formation in risk groups such as former smokers. The identification of ERK1/2 phosphorylation as an important event in SCC growth inhibition identifies it as a potential biomarker for n-3 PUFAs action *in vivo* but may also be informative in the identification or development of n-3 PUFAs that are more effective chemopreventive agents than EPA.

Finally, drugs that inhibit the EGFR such as cetuximab are currently undergoing phase II trials for the locoregional control of head and neck SCC (53), and n-3 PUFA supplements are often given to such patients post-operatively (54). Therefore, our results showing a potential antagonism between n-3 PUFAs and EGFR antagonists suggest that such trials should take this into account.

## Supplementary material

Supplementary Figures 1–3 can be found at http://carcin.oxfordjournals.org/

## Funding

Medical Research Council.

## Supplementary Material

Supplementary Data

## Abbreviations:

ANOVA: analysis of variance
DCF: 2′,7′-dichlorodihydrofluorescein diacetate
DHA: docosahexaenoic acid
EGFR: epidermal growth factor receptor
EPA: eicosapentaenoic acid
ERK 1/2: extracellular signal-regulated kinase 1/2
GAPDH: glyceraldehyde 3-phosphate dehydrogenase
JNK: c-jun N-terminal kinase
MTT: 3-(4,5-dimethylthiazol-2-yl)-2,5-diphenyltetrazolium bromide
p90RSK: p90 ribosomal S6 kinases
PBN: n-*tert*-butyl-α-phenylnitrone
PUFA: polyunsaturated fatty acid
ROS: reactive oxygen species
SCC: squamous cell carcinoma
TBS-T: Tris-Buffered Saline and Tween 20.
