# Supplementary material for: Omega-3 polyunsaturated fatty acids selectively inhibit growth in neoplastic oral keratinocytes by differentially activating ERK1/2
Source: Carcinogenesis. 2013 Jul 26;34(12):2716–25. doi: 10.1093/carcin/bgt257 (PMC3845892; doi:10.1093/carcin/bgt257)
Supplement: Supplementary Data [file supp_34_12_2716__index.html]

Omega-3 polyunsaturated fatty acids selectively inhibit growth in neoplastic oral keratinocytes by differentially activating ERK1/2 — Omega-3 polyunsaturated fatty acids selectively inhibit growth in neoplastic oral keratinocytes by differentially activating ERK1/2 — Omega-3 polyunsaturated fatty acids selectively inhibit growth in neoplastic oral keratinocytes by differentially activating ERK1/2 — Supplementary Data 

# Omega-3 polyunsaturated fatty acids selectively inhibit growth in neoplastic oral keratinocytes by differentially activating ERK1/2

## Supplementary Data

Data files

**Files in this Data Supplement:**

- Supplementary Data - Supplementary Data
