# Supplementary material for: Omega-3 polyunsaturated fatty acids selectively inhibit growth in neoplastic oral keratinocytes by differentially activating ERK1/2
Source: Carcinogenesis. 2013 Jul 26;34(12):2716–25. doi: 10.1093/carcin/bgt257 (PMC3845892; doi:10.1093/carcin/bgt257)
Supplement: Supplementary Data [file supp_bgt257_Nikolakopoulou_Supplementary_Figures_and_Legends_revised.doc]

**(a)**

**(b)**

**(c)**

**(d)**

**Supplementary Figure 1. The FACS profiles of the Annexin V assays conducted after n-3 PUFA treatment of SCC-25 cells.**

Representative flow cytometry plots for Annexin V-FITC/DAPI for cellular viability. The scatter plot (a) is shown for the untreated control where SSC=side scatter and FSC= forward scatter and the fluorescence plots are shown for each sample (b, c, d). SCC-25 cells were used in this example. Untreated control cells are shown in (b). Cells were treated with DHA 5 μM (c) and EPA 5 μM (d) for 48 h. Q3 shows the viable cell population (Annexin V -ve, DAPI -ve), Q4 shows early apoptotic cells (Annexin V +ve DAPI –ve), Q2 shows late apoptotic and necrotic cells (Annexin V +ve, DAPI +ve) and Q1 shows only necrotic cells (Annexin V –ve, DAPI +ve).

The figure illustrates a typical experiment from Figures 2a-b.

**
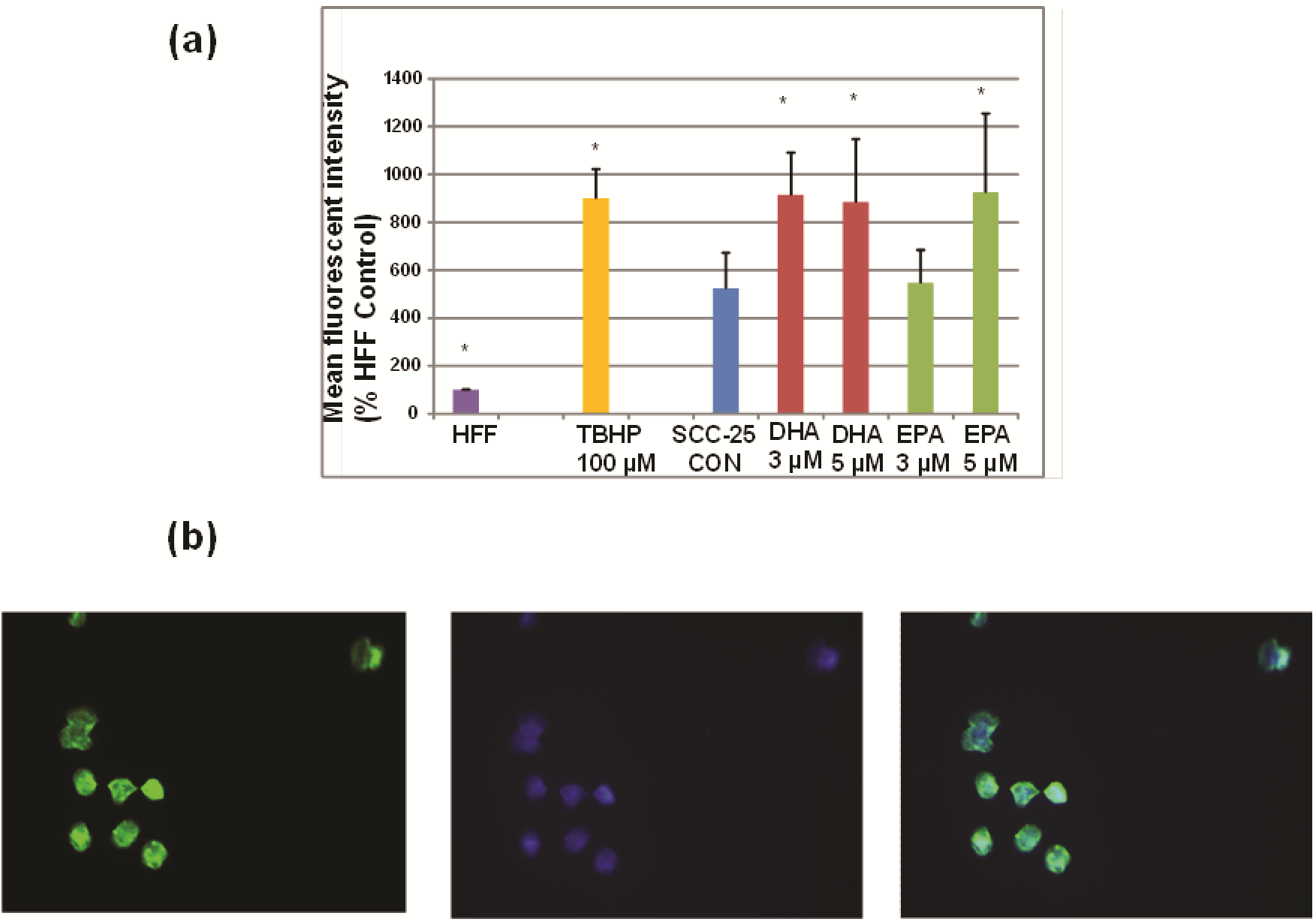
**

**Supplementary Figure 2. The effect of n-3 PUFAs on oxidative-damaged DNA as detected by 8-oxo-dG**

(a) The graph shows the mean fluorescence intensity of SCC-25 cells following staining for 8-oxo-dG after treatment with the selective (3 µM) and less selective (5 µM) doses of DHA and EPA for 16 hours. The lower dose of EPA did not induce 8-oxo-dG lesions but its higher dose and both doses of DHA induced levels of 8-oxo-dG comparable to the TBHP-treated positive controls. The fibroblast line, HFF, showed very low levels of 8-oxo-dG staining and served as a negative control in all experiments. The results are the means of 3 independent experiments +/- standard deviation. * is significantly different from the mean value of the untreated SCC-25 control (* = p<0.05 as measured by one-way ANOVA followed by post-hoc Bonferroni test).

(b) Representative images of SCC-25 cells staining, strongly positive with the 8-oxo-dG antibody. The SCC-25 cells were incubated with EPA 10 μM for 16 hours prior to staining. The left hand panel shows 8-oxo-dG staining in green, the middle panel the nuclear staining with Hoechst 33258 in blue and the right hand panel an overlay of the two.

**Supplementary Figure 3. The effect of the anti-oxidant PBN on the sensitivity of SCC-25 cells to n-3 PUFA-induced growth inhibition.**

The results show the effect of 600 µM (red) and 800 µM (green) doses of the anti-oxidant PBN on the growth inhibitory effect of 3-10 µM DHA (a) and EPA (b) as compared to the controls (blue) and as measured by the MTT assay. The results show that at doses of n-3 PUFAs that cause considerable DCF fluorescence (Figure 4) and oxidative damage to the DNA (Supplemental Figure 2) an anti-oxidant had no effect on n-3 PUFA-induced growth inhibition.
